# One Flu for One Health

**DOI:** 10.3201/eid1604.091593

**Published:** 2010-04

**Authors:** Ilaria Capua, Giovanni Cattoli

**Affiliations:** World Organisation for Animal Health, United Nations Food and Agriculture Organization, and National Reference Laboratory for Newcastle Disease and Avian Influenza, Legnaro, Italy; World Organisation for Animal Health Collaborating Centre for Diseases at the Human Animal Interface, Legnaro

**Keywords:** Influenza, genetics, viruses, zoonoses, globalization, global health, One Health, expedited, letter

**To the Editor:** The emergence and spread of influenza A pandemic (H1N1) 2009 virus from the animal reservoir to humans raise questions about the future approach to influenza virus infections. The scientific community has evidence demonstrating that influenza virus genes migrate across continents and animal species and assemble themselves in combinations that are a threat to animal and human health, resulting in panzootics like that caused by influenza A virus (H5N1) or pandemics like that caused by pandemic (H1N1) 2009 virus. The latter virus emerged from the animal reservoir, containing a unique combination of genes donated by viruses originating from from 3 species and 2 hemispheres. In a globalized environment, mapping gene movement across species and national borders and identifying mutations and gene constellations with pandemic potential or virulence determinants are essential to enact prevention and control strategies at a global level. This conclusion is in agreement with, and possibly the best example of, the One Health (http://un-influenza.org/node/2341) vision: a multidisciplinary collaborative approach to improving the health of humans, animals, and the environment. One Health is endorsed by the United Nations Food and Agriculture Organization, the World Organisation for Animal Health, and the World Health Organization.

Vast improvements in capacity building have been achieved as a result of the influenza A (H5N1) global crisis. Thousands of viral isolates with zoonotic potential have been obtained through surveillance efforts, although the genetic information has not been exploited fully. In addition, investigating how influenza viruses circulate in certain species, including dogs, pigs, and horses, has been neglected. This neglect is evidenced by the fact that, at the time of this writing, GenBank contained 4,001 full genome sequences of influenza viruses isolated from humans, 2,590 of viruses isolated from birds, and only 325 from swine, 85 from horses, 2 from mink, 4 from dogs, 2 from cats, 2 from tigers, and 3 from seals.

We invite donors and international agencies to invest in a novel approach to influenza virus infections, to abandon prefixed compartments linked to geographic origin or species of isolation, and to analyze the influenza gene pool as one entity. We propose capitalizing on existing achievements and investments to develop an international network and a permanent observatory, which will improve our understanding of the dynamics of the influenza virus gene pool in animals and humans. A greater understanding will generate important information to support both public and animal health. Ideally, a small consortium, including representatives of major international organizations, could take leadership and liaise with major institutions involved in influenza surveillance and research to develop a feasibility study and roadmap to achieve this goal. The One Flu initiative could result in international synergies, the bridging of gaps between medical and veterinary scientists, permanent monitoring of virus evolution and epidemiology, and the best exploitation of investments in capacity building. Above all, this collaboration could be a challenge and opportunity to implement the One Health vision, and possibly act as a model for other emerging zoonotic diseases.

